# The C2 Domain of PKC‑δ as a Dominant-Negative Modulator of Breast Cancer Cell Survival and Chemosensitivity

**DOI:** 10.1021/acsomega.5c08707

**Published:** 2025-12-15

**Authors:** Rasha Khader, Lodewijk V. Dekker

**Affiliations:** School of Pharmacy, Biodiscovery Institute, 6123 University of Nottingham, Nottingham NG7 2RD, U.K.

## Abstract

Mounting evidence implicates Protein Kinase C-δ (PKC-δ) in breast cancer progression and therapy resistance. PKC-δ is activated by the second messenger diacylglycerol or by proteolytic cleavage, both of which expose the kinase’s catalytic site and allow substrate phosphorylation. Furthermore, the C2 domain of PKC-δ regulates the kinase by mediating intra and intermolecular protein–protein interactions. Here, we investigated the autonomous effects of the PKC-δ C2 domain in two breast cancer cell lines, representing hormone-dependent and triple-negative breast cancer. A myc-tagged PKC-δ C2 domain (myc-δC2) was stably overexpressed in MCF-7 and MDA-MB-468 cells, and its effects on cell viability, apoptosis, and proliferation were assessed. myc-δC2 expression reduced cell viability and increased apoptosis in both cell lines. In MCF-7 cells, but not in MDA-MB-468 cells, G2/M arrest and increased cell size were observed upon myc-δC2 expression. Under oxidative (H_2_O_2_) and genotoxic (etoposide) stress, myc-δC2 expression sensitized cells differently in the two cell lines: MCF-7 cells showed consistent sensitization, whereas in MDA-MB-468 cells, sensitization was observed only at higher stress levels or after dasatinib pretreatment. These results indicate a cell line-dependent pro-death role for the isolated PKC-δ C2 domain, highlighting that modulation of this domain, or its use as an autonomous pro-apoptotic agent, may offer new therapeutic avenues in breast cancer.

## Graphical abstract

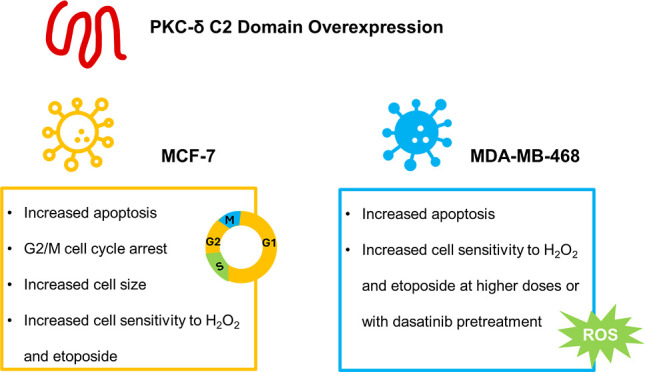

## 1 Introduction

Protein kinase C (PKC) is a family of serine/threonine kinases that regulates processes such as cell proliferation and motility.^1,2^ PKCs are classified into classical, novel, and atypical isoforms.^1,2^ Generally, PKC activation involves a conformational change exposing the substrate-binding site.^3^ For activation, classical PKCs require diacylglycerol and calcium binding, novel PKCs require diacylglycerol binding, while membrane proteins recruit atypical PKCs.^4^ Specific regions in the regulatory domain mediate these activation steps.^5,6^ For certain PKCs, a noncanonical activation occurs via proteolytic cleavage to release a constitutively active catalytic fragment.^7,8^

Novel PKC-δ contains a diacylglycerol-binding C1 domain and a C2 domain (Figure 1), which, unlike that in classical PKCs, does not bind Ca^2+^.^9^ Instead, it has protein binding capabilities, including with SMAC, GAP43, actin, and annexin V (see Table S1 for abbreviations).^10–14^ The C2 domain is a critical regulator of PKC-δ activity.^7,14,15,200^ Under oxidative (H_2_O_2_) or genotoxic stress (etoposide), the C2 domain Tyr64 phosphorylation exposes the nuclear localization signal, allowing importin-α/Hsp90-mediated nuclear translocation and apoptosis.^7,8,16^ Notably, it is also a phosphotyrosine-binding domain, recognizing phosphotyrosines in an aromatic context; for example, it may dock intramolecular phosphorylated Tyr334, which flanks a caspase-3 cleavage site implicated in generating a constitutively active catalytic fragment that drives nuclear translocation.^7,8,15,17^

**Figure 1:**
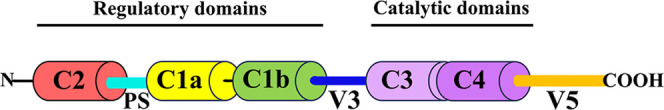
Simplified 2D structure of PKC-δ. PKC-δ is organized into regulatory and catalytic domains separated by a hinge region (V3 domain), which also serves as the caspase-3 cleavage site. The regulatory region includes the C2 domain, a phosphotyrosine-binding module, and tandem zinc finger C1 subdomains that bind diacylglycerol and phorbol esters to initiate kinase activation. The catalytic region consists of the C3 domain, containing the ATP-binding pocket, and the C4 domain, which provides the substrate-binding site. In the inactive state, the pseudosubstrate (PS) sequence occupies the substrate-binding site to maintain kinase inactivity. The C-terminal V5 domain harbors the nuclear localization signal as well as the hydrophobic and turn motifs critical for full kinase regulation.

PKC-δ has been implicated in breast cancer (BC) progression and therapy response.^18–21^ BC is a heterogeneous disease, classified into molecular subtypes based upon the expression of certain markers, including hormone receptors, and HER2.^22^ As such, its molecular subtypes include Luminal A, Luminal B, HER2-enriched, and triple-negative BC.^22^ Clinically, PKC-δ overexpression in ER^+^ BC correlates with improved endocrine therapy response,^23^ and it was linked to increased tamoxifen resistance.^24^

Preclinical studies indicate context-dependent roles of PKC-δ in BC.^13,19–21,25^ In MCF-7 (luminal A model), PKC-δ knockdown or inhibition with Rottlerin reduced proliferation,^19,21^ whereas overexpression increased apoptosis, seemingly engaging pro-apoptotic pathways.^20^ In triple-negative MDA-MB-468, PKC-δ was phosphorylated in a complex pattern unrelated to mezerein-induced growth inhibition,^25^ yet its overexpression sensitized these SMAC-mimetic-resistant cells to SMAC mimetics.^13^

While catalytic inhibition of PKC-δ is one therapeutic avenue, the role of its C2 domain in the regulation of this kinase suggests possible alternative ways to manipulate this kinase. However, data on manipulating PKC-δ via the C2 domain, especially in clinically relevant BC models, remain limited.

Here, we applied a dominant-negative strategy to assess whether interfering with the C2 domain affects hormone-dependent and triple-negative BC cells. We hypothesized that overexpressing the isolated C2 domain would sequester binding partners, disrupting endogenous PKC-δ C2 domain function. Our findings show that C2 domain overexpression profoundly alters cell viability, apoptosis, and treatment sensitivity, highlighting its substantial contribution to PKC-δ activity. Targeting PKC-δ at the C2 domain level, or exploiting the isolated domain as a pharmacological agent, may present novel therapeutic opportunities in BC.

## 2 Methods

### 2.1 Statistical Analysis

Data distribution was assessed using Shapiro–Wilk normality tests (α = 0.05). Normally distributed data (*P* > 0.05) were analyzed using one-way ANOVA with Tukey’s multiple comparisons test for single-time point experiments or two-way ANOVA (mixed-effects model) with Sidak’s multiple comparisons for time-course analyses. Non-normal data (*P* ≤ 0.05) were evaluated using the Friedman test with Dunn’s multiple comparisons test. All analyses were performed using GraphPad Prism 9 (GraphPad Software, San Diego, CA, USA) with statistical significance set at *P* < 0.05.

### 2.2 Cell Culture

Two BC cell lines, MCF-7 and MDA-MB-468, were grown in Minimum Essential Media Eagle, supplemented with 10% fetal bovine serum, 1% l-glutamine (200 mM), and 1% Penicillin streptomycin (10,000 U/ml). Cells were incubated at 37 °C and 5% CO_2_. All reagents and suppliers are listed in Table S2.

### 2.3 Transfection and Growing Stably Transfected Cell Lines

Cells were either transfected with the vector plasmid, pIRESneo2, or with myc-δC2-pIRESneo2 plasmid developed in-house as described in ref 26 to overexpress PKC-δ C2 domain (myc-δC2). Transfected cells were selected using Geneticin (G418, 400–800 μg/mL) and maintained under continuous antibiotic selection (G418, 300–350 μg/mL). Single colonies were isolated using cloning discs and expanded into stable cell lines (see Methods S1 for details).

### 2.4 Cell Lysate and Protein Quantification

Cells were seeded at 5 × 10^5^ cells/well in 6-well plates and were incubated until they reached confluency. Cells were lysed with 1x RIPA with protease and phosphatase inhibitors cocktail for 30 min on ice. Lysates were sonicated in water (3 cycles: 1 min on/1 min off) and centrifuged (17,000 RCF, 4 °C, 20 min). Protein concentration was quantified using Bradford assay (see Methods S2 for details). Protein expression was detected by Western blotting according to the protocol outlined in (ref 27) using anti-myc antibody (3:1000, 1 h, RT) and anti-GAPDH antibody (1:1000, overnight, 4 °C). Densitometry analysis was performed using ImageJ v1.54 (National Institutes of Health, Bethesda, MD, USA). Band intensities were quantified as integrated density values and normalized to GAPDH, which was used as the loading control.

### 2.5 Counting

Cells were counted weekly for over a year. Cells were seeded at 5 × 10^4^ cells/well in 6-well plates and incubated for 1 week, then harvested, homogenized using standard 23-gauge needles, and counted using equal volumes of trypan blue and a hemocytometer.

### 2.6 Tetrazolium Reduction Viability Assays (MTT Assays)

Cells were seeded at 4 × 10^3^ cells/well in 96-well plates and incubated for 24–144 h. Viability was assessed by incubating with 0.2% MTT (3 h), followed by DMSO solubilization. Absorbance (570 nm) was normalized to *T_0_* (3 h postseeding baseline). For detailed protocol, see Methods S3.

### 2.7 Treatment

Cells were seeded at 4 × 10^3^ cells/well in 96-well plates. After 24 h, they were treated with H_2_O_2_ (100 nM or 1 μM) or etoposide (100 μM or 200 μM). For pretreatment experiments, cells received 1 μM dasatinib 1 h prior to treatment. After 48 h, viability was assessed by MTT assay and normalized to untreated controls.

### 2.8 Apoptosis

Apoptosis was quantified using FITC Annexin V/PI staining. Cells were seeded at 1.1 × 10^5^ cells/well in 24-well plates for 48 h, then harvested, washed, and resuspended in binding buffer containing 5 μL of FITC Annexin V and 5 μL PI (full protocol is in Methods S4). After 15 min RT incubation in the dark, samples were analyzed by spectral flow cytometry (Sony ID7000, Sony Biotechnology, San Jose, CA, USA) and processed with Kaluza software (Beckman Coulter, Brea, CA, USA).

### 2.9 Cell Cycle Analysis

Cells were harvested at 24, 48, and 72 h, fixed in 70% ethanol (30 min, ice), and stained with PI (60 μM) and RNase A (20 μg/mL) in PBS. Samples were analyzed by spectral flow cytometry (Sony ID7000) and processed with Kaluza software (see Methods S5 for full protocol and gating details).

### 2.10 Cell Imaging

Following the same seeding and harvesting protocol as apoptosis assays, cells were washed twice with cold PBS and resuspended in 50 μL ice-cold PBS. Cells were imaged (ImageStream Mk II, Amnis Corporation, now part of Luminex Corporation, Seattle, WA, USA) 24–72 h postseeding, and morphometrics (diameter, area, circularity) were quantified using default gating in IDEAS 6.2 (Luminex Corporation, Austin, TX, USA). Data outputs included population statistics (mean, median, mode) and event counts (see Methods S6).

## 3 Results

Cells were transfected with the pIRESneo2-myc-δC2 plasmid to overexpress the C2 domain of PKC-δ (δC2 stable cell lines) or with pIRESneo2 vector plasmid (Vector stable cell lines). Following colony selection, time point zero was defined as the first time each transfected cell line reached confluency in T-75 flasks; all subsequent time points in this manuscript refer to weeks after this point. Accordingly, two independent MCF-7 Vector clones (labeled Vector.1 and Vector.2) and two MCF-7 δC2 clones (labeled δC2.1 and δC2.2) were established and discussed here; the same was done for MDA-MB-468.

Only statistically significant *P* values (*P* < 0.05) are shown; the absence of a *P* value indicates a nonsignificant result.

### 3.1 Myc-δC2 Expression and Initial Observations

Initial expression levels (4 weeks after time point zero) were quantified by Western blotting using an anti-myc antibody (Figure 2). For MCF-7 cells, clone δC2.1 showed 70% higher expression of myc-δC2 than clone δC2.2. For MDA-MB-468 cells, expression levels of myc-δC2 were 10% higher in clone δC2.1 than in δC2.2. We noticed expression levels then declined in all cell lines to undetectable levels, except in MCF-δC2.1, which maintained high expression levels throughout its lifespan. Expression levels eventually recovered in both MDA-MB-468 δC2 cell lines, but not in MCF-7 δC2.2.

**Figure 2:**
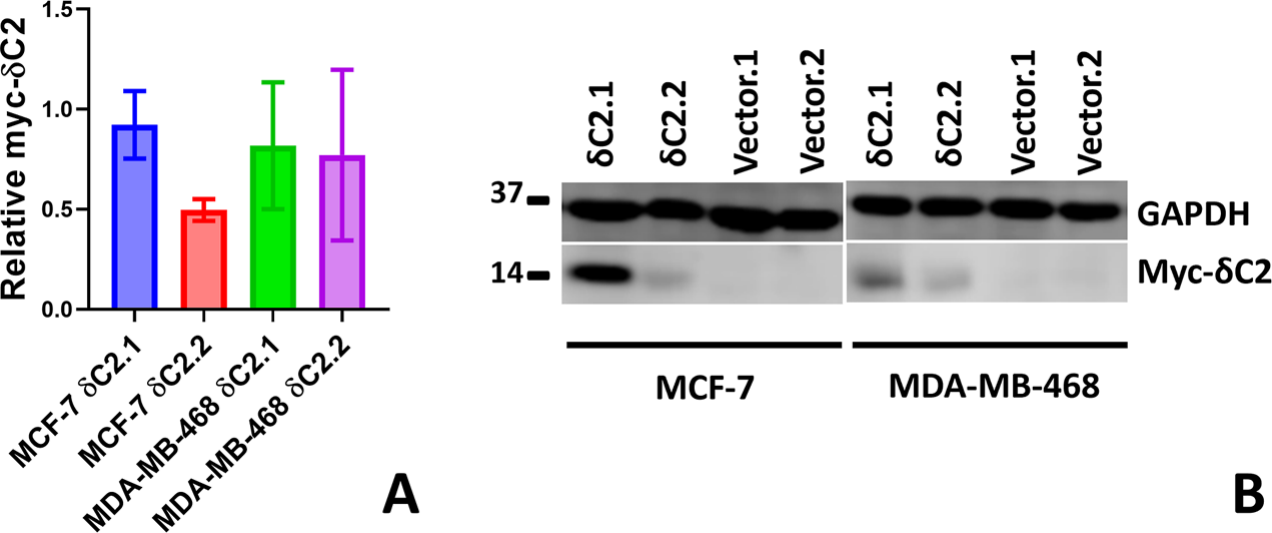
Myc-δC2 expression in stable cell lines. (A) Quantification of myc-δC2 levels at weeks 4, 6, and 10, normalized to the loading control GAPDH. Bars represent mean ± SEM of *N* = 3 biological replicates. (B) Representative blot at week 4, confirming stable transfection. Full, uncropped blots are available in Figure S2.

Vector colonies of both cell lines appeared earlier in transfection wells and grew and expanded noticeably faster than δC2 clones. Moreover, after MCF-7 colonies were stably growing in T-75 flasks, Vector clones reached confluency within 5–6 days, compared to 7–9 days for δC2 clones.

### 3.2 Myc-δC2 Expression Affects Cell Viability

Early viability assays were conducted on all four transfected cell lines for both cell types at 24, 48, 72, and 144 h (Figure 3). Expression levels of myc-δC2 were detectable in all δC2 clones and in both cell types (Figure 2). For both cell types, viability was significantly lower in δC2 clones compared to Vector clones at all time points (*P* < 0.0001; in both cell types), suggesting an inverse correlation between myc-δC2 expression and cell viability.

**Figure 3:**
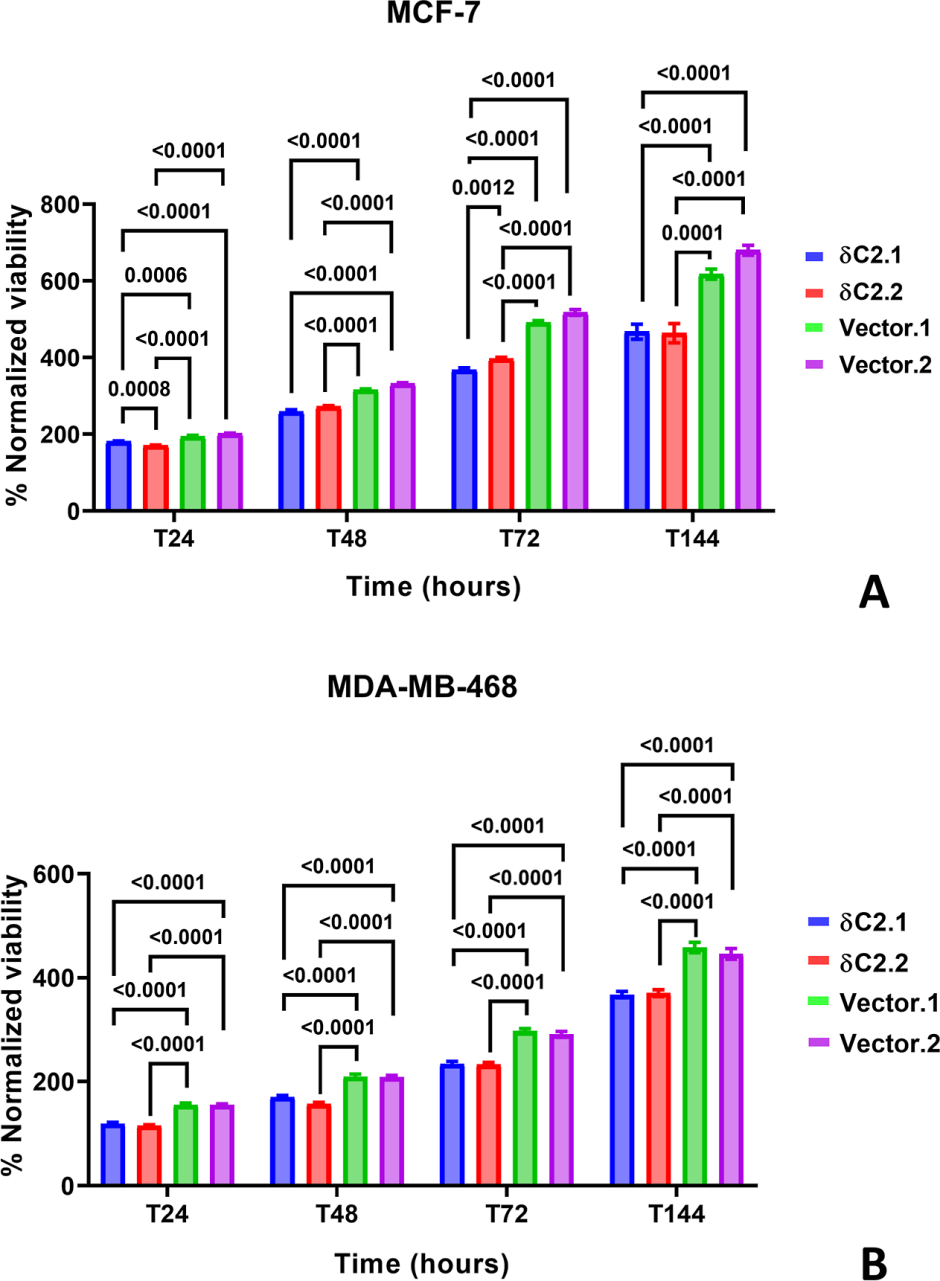
High levels of myc-δC2 reduce viability in (A) MCF-7 and (B) MDA-MB-468 cells at 24, 48, 72, and 144 h. Viability was normalized to *T*_0_ values. Statistical significance was determined using a two-way ANOVA with a full mixed model and Sidak’s multiple comparisons. Bars represent mean ± SEM of *N* = 15 independent biological replicates; each independent biological replicate was the average of 3 technical replicates.

During this study, we noticed that myc-δC2 expression levels fluctuated over time. This created an opportunity to further establish the inverse correlation between myc-δC2 expression and cell viability. δC2 clones with negligible myc-δC2 expression levels were expected to behave similarly to Vector clones. Twenty-eight weeks after establishing the stable clones, myc-δC2 expression was readily detectable in MCF-7 δC2.1 (Figure 4A), while MCF-7 δC2.2 exhibited undetectable levels. Trypan-blue counting assays showed that the viability of δC2.1 was significantly lower than that of Vector.1, and Vector.2 (*P* < 0.0001, Figure 4B). No significant difference in viability was seen between δC2.2 and Vector clones. Thus, the viability of MCF-7 cells was consistently inversely correlated with the expression levels of myc-δC2 in these cells.

**Figure 4:**
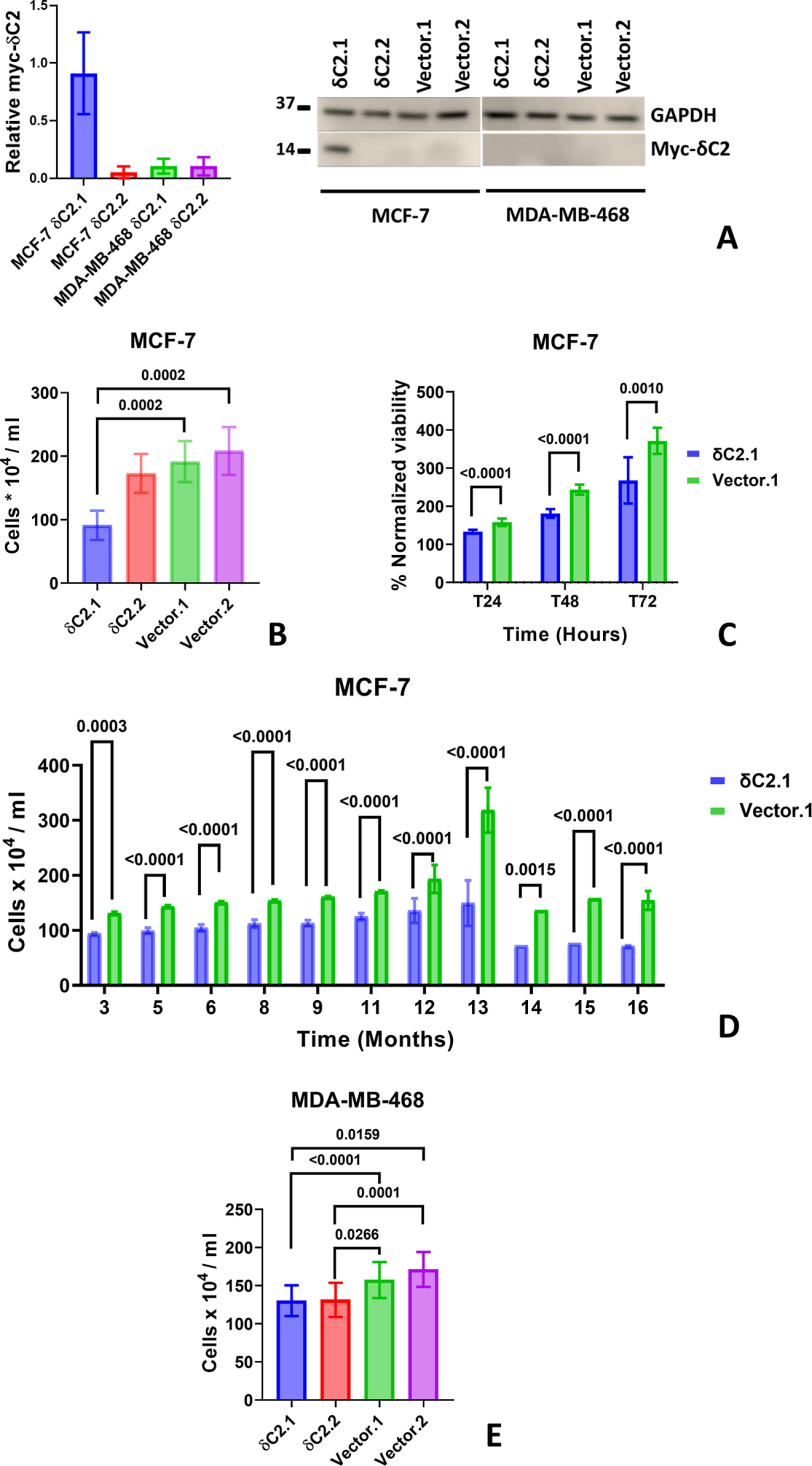
Viability analysis under declined myc-δC2 expression. (A) Relative myc-δC2 expression over the experimental period for panels (B–E), quantified by Western blotting and normalized to GAPDH (loading control). Among the stable clones, only MCF-7 δC2.1 retained consistently higher expression, whereas the other clones showed very low, intermittently undetectable levels. Bars represent mean ± SEM of *N* = 3 biological replicates. Full, uncropped blots are available in Figure S3. (B) MCF-7 cell count over 10 weeks. Bars represent mean ± SEM of *N* = 10 biological replicates. (C) MTT viability of MCF-7 δC2.1 compared to Vector.1 over 10 weeks and at three time points (normalized to *T*_0_). Bars represent mean ± SEM of *N* = 10 independent biological replicates; each independent biological replicate was the average of 3 technical replicates. (D) Long-term growth of MCF-7 δC2.1 compared to Vector.1. Bars represent mean ± SEM across *N* = 11 months of independent trials. Each monthly value reflects the average of 1–5 independent biological replicates performed that month (with ≥1 biological replicate per week). (E) MDA-MB-468 cell count over 12 weeks. Bars represent mean ± SEM of *N* = 12 independent biological replicates. Analysis was done using (B,E) Friedman test with Dunn’s multiple comparisons and (C,D) two-way ANOVA with a full mixed model and Sidak’s multiple comparisons.

To confirm this further, we focused on δC2.1, which maintained detectable expression, and compared its viability to Vector.1 over a ten-week time window using MTT assays. δC2.1 viability was significantly lower at all time points (*P* = 0.0005, Figure 4C). To account for changes in expression levels over time, MCF-7 δC2.1 and Vector.1 were monitored weekly for over a year (Figure 4D). δC2.1 consistently showed significantly lower viability than Vector.1 at all time points (*P* < 0.0001). Overall, this confirmed the inverse correlation between myc-δC2 expression and cell viability in MCF-7 cells.

We also noticed that myc-δC2 expression in both MDA-MB-468 δC2 clones was below detection in weeks 28–36 after establishing the cell lines, and hypothesized that these cells would now behave similarly to Vector clones. Viability in MDA-MB-468 δC2 clones during this time window was reduced compared to the Vector counterparts (*P* < 0.0001, Figure 4E), but the reduction in viability was less prominent than in the MCF-7 δC2.1 (Figure 4B). This may be due to small levels of myc-δC2 expression below the detection threshold of our Western blots, or possibly due to prolonged downstream consequences of prior myc-δC2 expression. Subsequent analysis indicated that myc-δC2 expression re-emerged in these cell lines, confirming that the myc-δC2-transfected DNA had not been deleted from the cells (Figure 5A).

**Figure 5:**
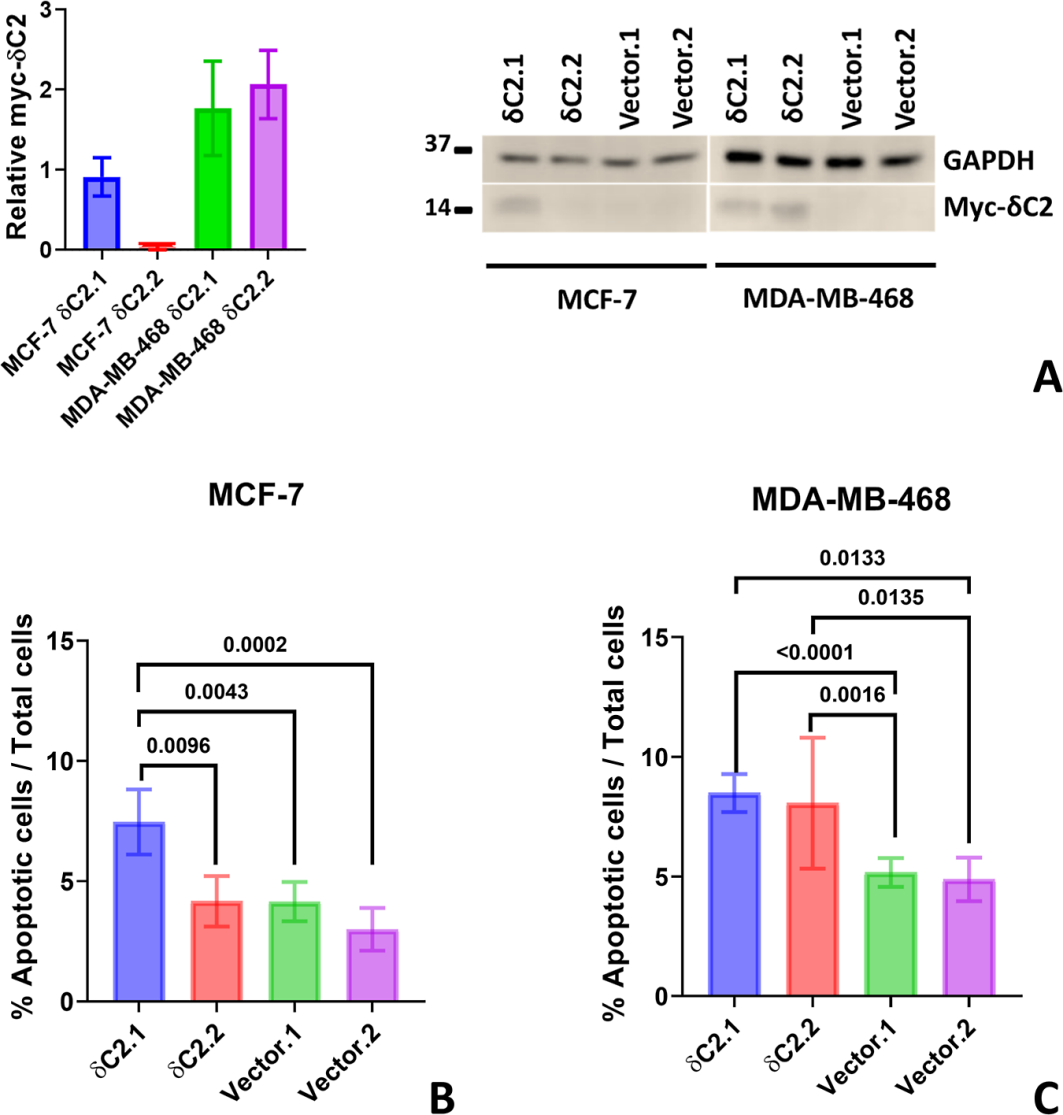
Apoptotic effects of myc-δC2. (A) Relative myc-δC2 expression during the experimental period corresponding to panels (B,C), quantified by Western blotting and normalized to GAPDH (loading control). Bars represent mean ± SEM of *N* = 3 biological replicates. Full, uncropped blots are available in Figures S4 and S5. (B,C) Total apoptosis (early + late apoptosis) in transfected cell lines after 48 h of incubation: (B) MCF-7 (Bars represent mean ± SEM of *N* = 7–11 biological replicates) and (C) MDA-MB-468 (Bars represent mean ± SEM of *N* = 8–20 biological replicates). Data were analyzed using one-way ANOVA with a full mixed model and Tukey’s multiple comparisons.

### 3.3 Myc-δC2 Expression Increases Apoptosis

To understand the mechanism behind the myc-δC2-induced loss of viability, we performed apoptosis assays on the cell lines described above. Apoptosis was quantified at a time point when myc-δC2 expression was high in MCF-7 δC2.1 clone but nearly undetectable in MCF-7 δC2.2 (Figure 5A). Consequently, MCF-7 δC2.1 exhibited significantly higher levels of apoptosis compared to δC2.2, Vector.1 and Vector.2 (*P* = 0.0002, Figure 5B).

In MDA-MB-468 δC2 clones, expression of myc-δC2 at the time point of apoptosis analysis was readily detectable in both clonal cell lines (Figure 5A). This increase in myc-δC2 expression correlated with a significant elevation in apoptosis in both MDA-MB-468 δC2 clones compared to Vector clones (*P* < 0.0001, Figure 5C).

Thus, for both MCF-7 and MDA-MB-468 cells, the presence of myc-δC2 appears to uncover an apoptotic response, which may underlie the reduction in cell numbers observed before.

### 3.4 Myc-δC2 Expression is Associated with Cell Cycle Arrest in MCF-7 but Not MDA-MB-468

Since PKC-δ has been implicated in pro-proliferative responses in BC cells, we measured the effect of myc-δC2 expression on cell cycle progression. Cell cycle distribution was assessed by PI staining at 24, 48, and 72 h postseeding (Figure 6A). As cells were largely in lag phase at 24 h and 48- and 72 h profiles were similar, only 72 h data are presented.

**Figure 6:**
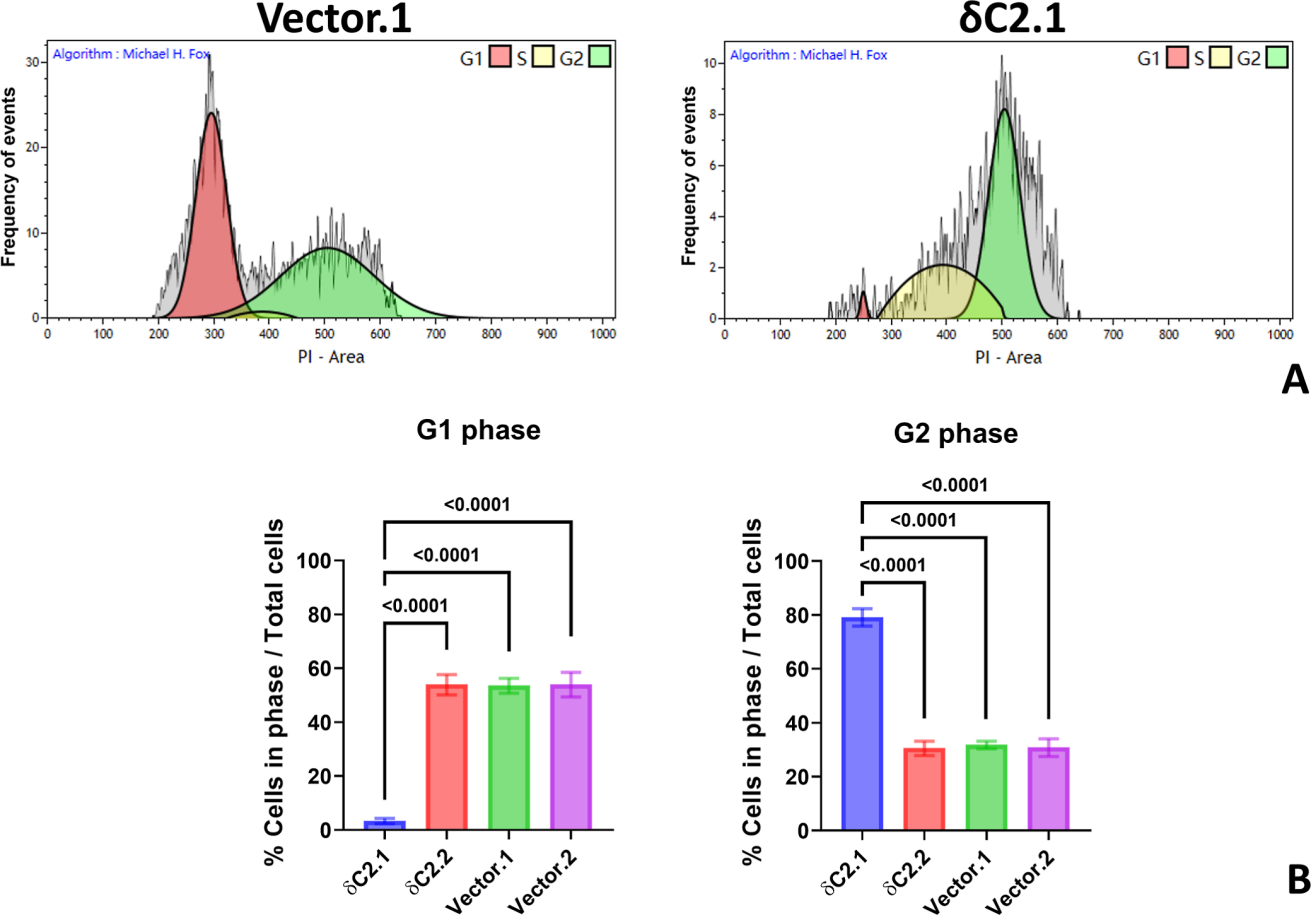
Cell cycle analysis of MCF-7 cells. (A) Representative cell cycle histogram of PI-stained δC2.1 and Vector.1 clones analyzed using Kaluza software with doublets discrimination applied. (B) Quantitative distribution of cell cycle phases (G1 and G2/M) across four transfected MCF-7 lines after 72 h incubation. Statistical significance was determined using one-way ANOVA with Tukey’s multiple comparisons. Bars represent mean ± SEM of *N* = 4 independent biological replicates; each independent biological replicate was the average of 3 technical replicates.

MCF-7 δC2.1 was the only MCF-7 line with detectable myc-δC2 throughout the assay period (Figure 5A). While Vector and δC2.2 clones showed normal cell cycle progression, MCF-7 δC2.1 cells were arrested in the G2 phase, accompanied by a significant depletion in the G1 phase (*P* < 0.0001, Figure 6B). Although the S-phase accumulation was increased, this change did not reach statistical significance. Therefore, in MCF-7 cells, myc-δC2 appears to be a cell cycle-arresting cytotoxic agent.

In contrast, MDA-MB-468 cells expressed high and comparable levels of myc-δC2 (Figure 5A), yet all four MDA-MB-468 clones displayed normal cell cycle profiles. The distribution across cell cycle phases was comparable between the δC2 and Vector clones (data not shown). Therefore, it appears that MDA-MB-468 cells respond differently to myc-δC2 expression than MCF-7 cells and that myc-δC2 expression is mainly pro-apoptotic without affecting the cell cycle.

### 3.5 Myc-δC2 Expression Increases Cell Size in MCF-7 but Not MDA-MB-468

Visual inspection under the microscope revealed that MCF-7 δC2 clones, particularly δC2.1, appeared larger and more readily identifiable. The size difference was also apparent during flow cytometric analysis of apoptosis assays, where FSC-SSC plots showed MCF-7 δC2.1 cells distributed broadly along the FSC axis, consistent with increased cell size.^28^

To confirm this, cell imaging flow cytometry (Figure 7A) was performed in parallel with the apoptosis and cell cycle assays. At that time point, myc-δC2 expression was high in MCF-7 δC2.1 and MDA-MB-468 δC2.1 and δC2.2 clones, and negligible in MCF-7 δC2.2 (Figure 5A).

**Figure 7:**
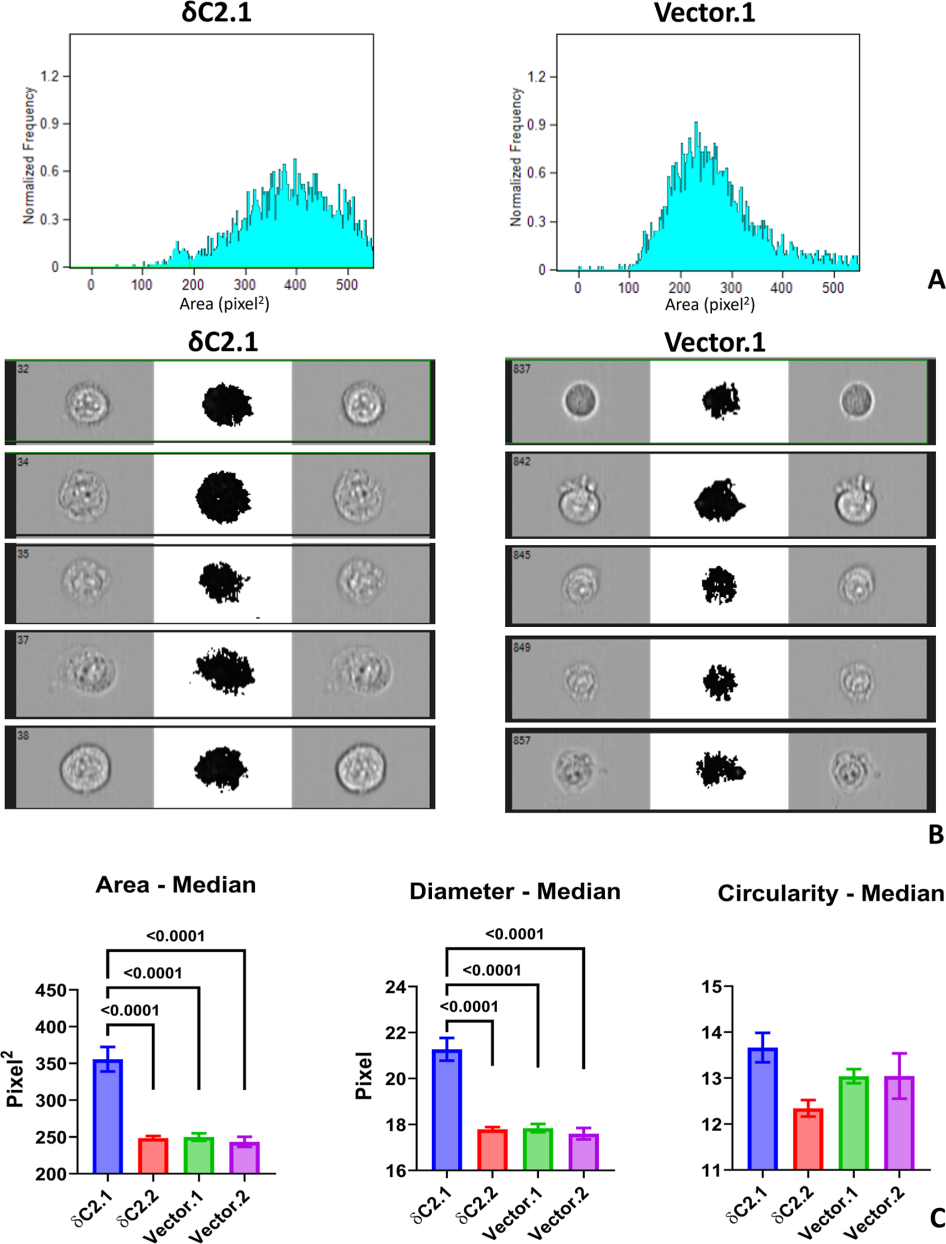
Morphological analysis of MCF-7 cells. (A) Representative IDEAS plots of the calculated surface area of δC2.1 (*n* = 10,000 cells, mean = 380 pixel^2^) compared to Vector.1 (*n* = 10,000 cells, mean = 250 pixel^2^). (B) Phase contrast images of single δC2.1 cells compared to Vector.1 cells at three different light intensities, demonstrating size differences. (C) Quantitative analysis of the physical features of all four transfected MCF-7 cell lines after 72 h of incubation. Statistical significance was calculated using one-way ANOVA with Tukey’s multiple comparisons. Bars represent mean ± SEM of *N* = 4 independent biological replicates; each independent biological replicate was the average of 2 technical replicates.

Images of 10,000 unstained cells were captured at three light intensities after 72 h of incubation. From these cells (Figure 7B), cell size (surface area and diameter) and shape (circularity) were quantified. MCF-7 δC2.1 cells exhibited a 50% increase in surface area and diameter compared to δC2.2 and Vector clones (*P* < 0.0001, Figure 7C). Circularity varied among lines but did not differ significantly.

Despite high myc-δC2 expression in MDA-MB-468 δC2.1 and δC2.2 (Figure 5A), no significant differences in cell size or morphology were detected compared to Vector clones (data not shown).

### 3.6 Myc-δC2 Overexpression Enhances Sensitivity to Oxidative and Genotoxic Stress

PKC-δ has been implicated in stress-induced responses through a mechanism that involves tyrosine phosphorylation on multiple residues.^7,8,16^ To assess the impact of myc-δC2 expression on stress sensitivity, MCF-7 and MDA-MB-468 δC2.1 and Vector.1 clones were treated with H_2_O_2_ (100 nM or 1 μM) or etoposide (100–200 μM) for 48 h. Expression levels of myc-δC2 at the time of treatment are shown in (Figure 5A). In MCF-7 cells, δC2.1 cells exhibited significantly reduced viability compared to Vector.1 at all tested concentrations (*P* = 0.0001 for H_2_O_2_, *P* = 0.0222 for etoposide, Figure 8A,B). To evaluate the contribution of tyrosine phosphorylation, cells were pretreated with dasatinib (1 μM, 1 h), a Src-family kinase inhibitor known to block PKC-δ phosphorylation at Tyr64 and other tyrosines.^29^ While dasatinib reduced cytotoxicity across all conditions, consistent with the known pro-apoptotic role of Tyr64 phosphorylation, δC2.1 cells remained significantly more sensitive than Vector.1 (*P* = 0.02–0.0001). These findings suggest that myc-δC2 enhances cell death through both tyrosine phosphorylation-dependent (dasatinib-sensitive) and -independent pathways.

**Figure 8:**
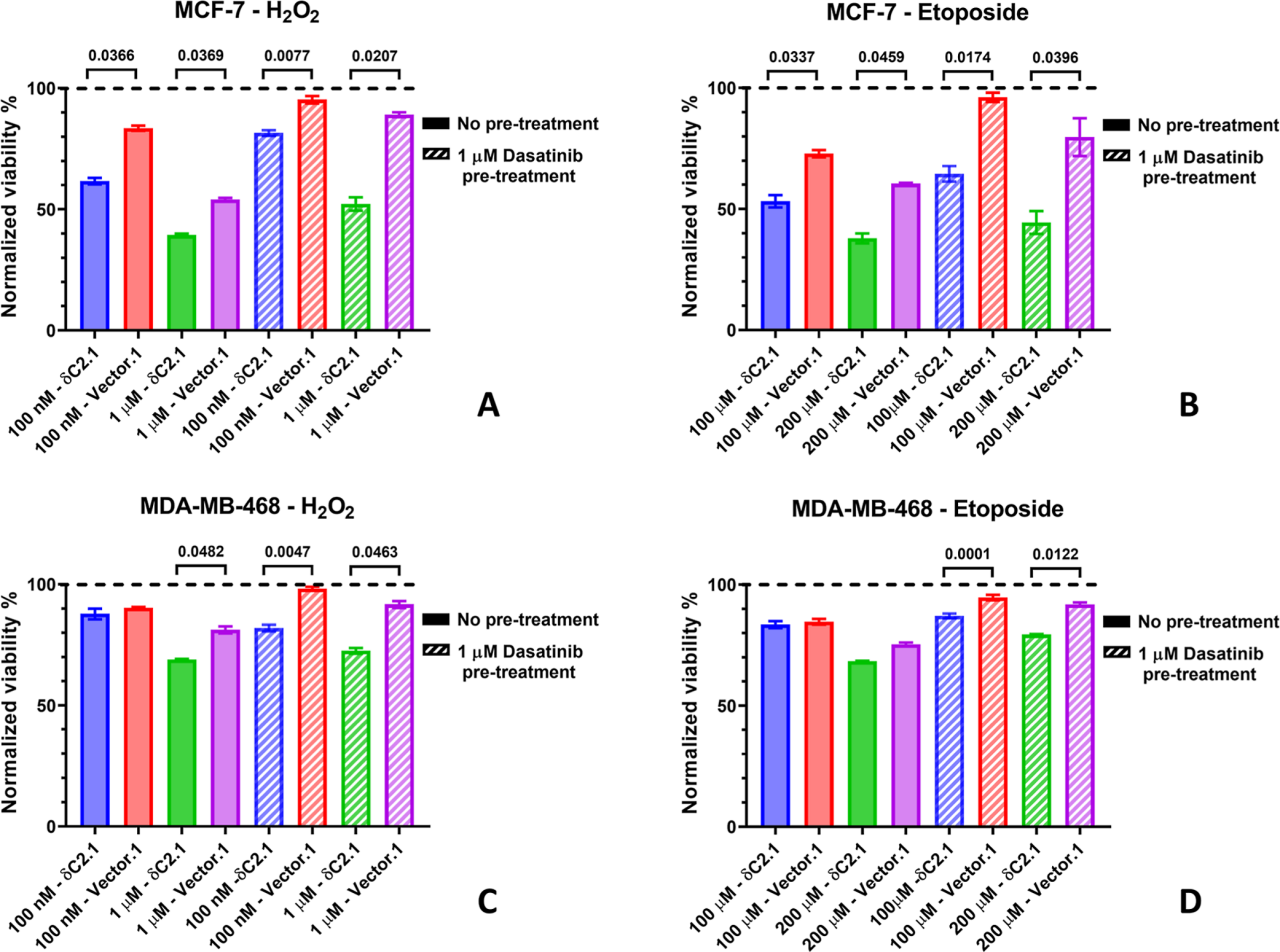
Myc-δC2 alters cell response to treatment. (A,B) MCF-7 and (C,D) MDA-MB-468 δC2.1 and Vector.1 clones were treated with (A,C) H_2_O_2_ (100 nM, 1 μM) and (B,D) etoposide (100 μM, 200 μM) for 48 h. Pattern bars indicate pretreatment with 1 μM Dasatinib for 1 h. Viability was normalized to untreated controls (dotted line) or pretreated controls. Statistical significance was determined using a one-way ANOVA test with Tukey’s multiple comparisons. Bars represent mean ± SEM of *N* = 3 independent biological replicates; each independent biological replicate was the average of 2 technical replicates.

In MDA-MB-468 cells, myc-δC2 expression had no measurable impact on viability at lower treatment doses (100 nM H_2_O_2_, 100 μM etoposide), but significantly increased sensitivity at higher concentrations (1 μM H_2_O_2_, 200 μM etoposide) (*P* = 0.0015 for H_2_O_2_, *P* = 0.0046 for etoposide, Figure 8C,D). Notably, dasatinib provided greater cytoprotection in Vector.1 than in δC2.1 (*P* = 0.001–0.005), indicating that myc-δC2 expression diminishes the efficacy of dasatinib rescue, likely by activating additional apoptotic pathways that are independent of tyrosine phosphorylation. However, these observations are based on a single inhibitor and should be interpreted in this context.

## 4 Discussion

### 4.1 Myc-δC2 Affects Cell Viability

PKC-δ C2 domain was overexpressed in two BC cell lines to assess its impact on cellular functions. It was hypothesized that overexpressing PKC-δ C2 domain would titrate out essential functional interactions of the endogenous PKC-δ C2 domain, thus affecting cell behavior. At the same time, the consequences of myc-δC2 expression in the cells could be interpreted pharmacologically by treating the isolated PKC-δ C2 domain as a standalone pharmacological agent.

The establishment of stable cell lines introduced additional complexity, as G418 selection was used to maintain integration of the pIRESneo2 vector. Cells initially exhibited high levels of myc-δC2 expression 4 weeks into selection, followed by an apparent progressive decline, perhaps due to epigenetic silencing or expansion of low-expressing clones under selective pressure.^30,31^ Interestingly, expression levels later rebounded in MDA-MB-468 cells, potentially reflecting epigenetic reactivation of previously silenced constructs or stochastic expansion of cells with more stable integration under continuous G418 selection.^30,31^

Myc-δC2 expression caused a significant reduction in cell viability in MCF-7 and MDA-MB-468 cells. In MCF-7, cell viability was significantly reduced via two mechanisms: increased apoptosis and reduced proliferation, whereas only apoptosis was observed in MDA-MB-468. Myc-δC2 expression in MCF-7 also increased cell size. Hence, the impact of myc-δC2 expression was more pronounced in MCF-7.

In MCF-7 δC2 clones, the decline in myc-δC2 expression was accompanied by a recovery of viability to levels comparable to Vector controls, and expression remained undetectable thereafter. In MDA-MB-468 δC2 clones, viability was significantly reduced even when myc-δC2 expression was not detectable by Western blotting, although the magnitude of reduction at that time point was smaller than observed in MCF-7.

### 4.2 Myc-δC2 Expression Increases Apoptosis

Myc-δC2 may potentially interact with native C2 domain ligands present in the cells, such as annexin V, SMAC, and phosphotyrosine-containing peptides.^14,15,32–35^ These interactions could modulate apoptosis through competitive inhibition or sequestration mechanisms. For example, the IAP-inhibitor SMAC not only maintains apoptosis by inhibiting the IAPs but has also been reported to bind the C2 domain of PKC-δ.^13,14,36^ Such binding might influence PKC-δ localization to the nucleus or mitochondria, potentially contributing to pro-apoptotic effects.^13,14^

Additionally, myc-δC2 may interact with phosphotyrosine-containing proteins. For instance, Hsp90 is activated by PKC-δ phosphorylation at Tyr313 and Tyr197,^37^ and ERK1/2 activation involves phosphorylation at several tyrosines (e.g., Tyr204, Tyr187).^35,38^ Binding of myc-δC2 to these phosphotyrosines could theoretically interfere with their function and downstream apoptotic pathways, which are known to involve PKC-δ in multiple cell lines.^19,20,39,40^

Moreover, the expressed myc-δC2 could potentially interact with the C1 subdomains of native PKC-δ or other PKC isoforms, thereby modulating their activity or apoptotic pathways.^41^ This domain has also been described to engage the catalytic^42^ and V5 domains to maintain kinase inactivity in other PKC isoforms,^43^ and to associate with membranes following PKC activation.^43,201^ While these interactions remain speculative, they represent plausible mechanisms by which myc-δC2 expression might influence apoptosis. The precise molecular mechanisms underlying these observations are currently under investigation.

It may be argued that expression of PKC-δ C2 domain could titrate out the function of other C2 domain-containing proteins; it should be noted that the PKC-δ C2 domain fold has its own unique features, with differences in topological environment, loop structures, and positioning of small helical insertions compared to the C2 domains of cPLA2, synaptotagmin and classical PKCs (discussed in ref 9). PKC-δ C2 domain has the highest homology with the C2 domain of PKC-θ and could potentially inhibit this isotype; however, expression levels of PKC-θ in MCF-7 and MDA-MB-468 cells are considered low.^19,44^

### 4.3 Myc-δC2 Expression Increases Cell Size of MCF-7

In MCF-7 cells, myc-δC2 expression significantly increased cell size. Cell size control is crucial, and cells that do not have the correct size are not allowed to continue the cell cycle.^45^ Cells first increase their size to double their genetic material, and later, they are checked for size and DNA content.^45^ It is possible that MCF-7 δC2 clones arrest at the G2 checkpoint due to abnormal size or disrupted DNA integrity.^35^ This was previously observed in yeast, where a DAF1 mutation reduced cell size, while the silencing of DAF1 increased cell size by 50%; both conditions altered the cell cycle progression.^46^ Additionally, cells monitor size through mTOR and protein kinase A pathways.^45,47^ These pathways control ribosome biogenesis, an indicator of the cellular nutritional status that correlates with cell size.^45,48^ The size increase may relate to mTOR signaling, which is functionally connected to PKC-δ via nuclear transport mechanisms.^47,49,50^ PKC-δ was also previously linked to cell size, as PMA treatment in cells overexpressing PKC-δ caused increased cell size and cell cycle arrest; no such response was observed in wild-type.^51^ Moreover, PKC-δ-controlled ERK1/2-invloved alteration of cell morphology was previously suggested.^52^ We are currently examining the downstream signaling events involved.

### 4.4 Myc-δC2 Overexpression Enhances Sensitivity to Stress

Our findings establish the PKC-δ C2 domain as a regulator of BC cell fate, modulating apoptosis and stress sensitivity via both phosphorylation-dependent and -independent mechanisms. In luminal MCF-7 cells, myc-δC2 expression uniformly enhanced sensitivity to both agents, consistent with its established role in promoting apoptosis and cell-cycle arrest. This suggests the C2 domain dominates stress response pathways in this cell type, potentially through its ability to both initiate death signaling and suppress survival mechanisms.

The triple-negative MDA-MB-468 model revealed a more complex, biphasic response. The absence of myc-δC2-mediated sensitization at lower doses suggests competitive interaction with endogenous PKC-δ survival signaling, while the increased sensitivity at higher concentrations indicates this buffering capacity becomes overwhelmed, allowing myc-δC2’s pro-apoptotic functions to prevail. This threshold effect may reflect differential activation of compensatory survival mechanisms in aggressive versus luminal subtypes.

Dasatinib inhibition experiments provided key mechanistic insights: blocking PKC-δ tyrosine phosphorylation reduced overall cytotoxicity in all cells, yet δC2 clones remained significantly more sensitive than Vector clones. These results suggest that myc-δC2 enhances treatment-induced cell death through both phosphorylation-dependent (dasatinib-sensitive) and -independent pathways. Importantly, because myc-δC2 expression alone also induces apoptosis in the absence of external stressors, it likely functions as both a standalone pro-apoptotic signal and a sensitizer to genotoxic and oxidative stress. However, it is important to note that these conclusions are based on a single inhibitor, dasatinib, and additional studies using other inhibitors would be needed to fully confirm these phosphorylation-dependent and -independent mechanisms. The limited rescue by dasatinib in δC2 clones further suggests that myc-δC2 expression engages apoptotic pathways beyond those regulated by phosphorylation, highlighting a dual mechanism that may be especially relevant under high-dose treatment conditions.

### 4.5 Research Contributions and Potential Applications

This study demonstrates that the C2 domain of PKC-δ, when introduced into two different BC cell lines, modulates cell survival, apoptosis, and stress sensitivity. In MCF-7 cells, it also induces cell cycle arrest and increases cell size. These findings suggest that the PKC-δ C2 domain can be viewed as a pharmacological tool to modulate cancer cell behavior and could be explored therapeutically. Furthermore, this study provides experimental evidence that a regulatory domain of PKC-δ can influence apoptotic and stress-response pathways, highlighting a previously underappreciated layer of kinase regulation.

PKC isotype-specific small-molecule inhibitors targeting the ATP-binding site have been reported for several PKC isoforms.^53^ For example, a triazole analogue of sotrastaurin has been developed as a small-molecule inhibitor of PKC-δ.^54^ In addition, the doxorubicin analogue AD198 has been suggested to activate the pro-apoptotic function of PKC-δ, possibly through actions on its C1 (diacylglycerol-binding) domain.^55^ Here, we show that overexpression or delivery of the PKC-δ C2 domain represents a protein-based strategy to trigger apoptosis and enhance chemosensitivity. Future therapeutic development could therefore exploit multiple avenues for manipulating PKC-δ, including ATP-binding site inhibition as well as C1 and C2 domain modulation.

## 5 Conclusion

The PKC-δ C2 domain links apoptotic control with cell-cycle and stress-response pathways in two BC cells. These findings provide proof-of-concept for domain-focused modulation of PKC-δ activity and highlight the need for in-depth validation of C2 domain-based or mimetic strategies in translational models to assess their therapeutic potential and selectivity.

## Supplementary Material



## Data Availability

All data supporting the findings of this study are available within the manuscript and the Supporting Information.

## Notes

Detailed experimental methods and reagent information (PDF)

## †

Department of Clinical Pharmacy, Faculty of Pharmacy, Jordan University of Science and Technology, P.O. Box 3030, Irbid 22110, Jordan
