# Supplementary material for: The C2 Domain of PKC‑δ as a Dominant-Negative Modulator of Breast Cancer Cell Survival and Chemosensitivity
Source: ACS Omega. 2025 Dec 15;10(51):62927–37. doi: 10.1021/acsomega.5c08707 (PMC12756763; doi:10.1021/acsomega.5c08707)
Supplement: Supplementary file 1 [file ao5c08707_si_001.pdf]

Supporting Information for:

The C2 Domain of PKC-δ as a Dominant-Negative Modulator of Breast Cancer Cell Survival and Chemosensitivity

Rasha Khader[*,†] and **Lodewijk V. Dekker***

School of Pharmacy, Biodiscovery Institute, University of Nottingham, Nottingham NG7 2RD, U.K.

* Email: rekhader@just.edu.jo

* Email: lodewijk.dekker@nottingham.ac.uk

Table S1. List of abbreviations.

Abbreviation	Definition
PKC-δ	Protein kinase C-δ
BC	Breast cancer
CMV	Cytomegalovirus
cPLA2	Cytosolic phospholipase A2
DMSO	Dimethyl sulfoxide
ER^+	Estrogen receptor-positive breast cancer
ERK1/2	Extracellular signal-regulated kinase 1/2
FBS	Fetal bovine serum
FSC	Forward scatter
G418	Geneticin
GAP43	Growth Associated Protein 43
GAPDH	Glyceraldehyde 3-phosphate dehydrogenase
HER2	Human epidermal growth factor receptor 2
Hsp90	Heat shock protein 90
IAP	Inhibitor of apoptosis protein
MEM	Minimum Essential Medium Eagle
mTOR	Mammalian target of rapamycin
MTT	Tetrazolium reduction viability assay
PI	Propidium iodide
PMA	Phorbol 12-myristate 13-acetate
RIPA	Radioimmunoprecipitation assay buffer
RNase A	Ribonuclease A
RT	Room temperature
SMAC	Second Mitochondria-derived Activator of Caspases
SSC	Side scatter

Table S2. Material supplier details.

Material	Supplier	Application
MCF-7 and MDA-MB-468 cell lines	ATCC, Manassas, Virginia, USA	Cell culture
Minimum essential media Eagle (MEM)	Sigma-Aldrich, Gillingham, UK	Cell culture
Foetal bovine serum	Sigma-Aldrich, Gillingham, UK	Cell culture – final concentration 10 % v/v
L-glutamine, 200 mM	Gibco, São Paulo, Brazil	Cell culture – final concentration 1 % v/v
Penicillin Streptomycin	Gibco, São Paulo, Brazil	Cell culture – final concentration 1 % v/v
pIRESneo2	Clontech (now Takara Bio Europe), Saint-Germain-en-Laye, France	Transfection
Lipofectamine 2000	Invitrogen, Lithuania	Transfection – final concentration 2.4% v/v final concentration
Geneticin	Gibco, São Paulo, Brazil	Selection
Cloning discs	SP Scienceware, Singapore	Colony transfer
RIPA, 10x	Thermo Scientific, Loughborough, UK	Cell lysates – 1x diluted in deionized water
Protease and phosphatase inhibitors cocktail tablets	Roche, Basel, Switzerland	Cell lysates – one tablet/10 ml 1x lysis buffer
Pierce Bradford assay reagent	Thermo Scientific, Loughborough, UK	Protein quantification
Anti-myc mouse monoclonal (9E10) antibody	EMD Millipore Corporation, Burlington, MA, USA	Western blotting – 3:1000 dilution in 5% BSA in TBST for 1 hour at RT
Anti-GAPDH rabbit monoclonal (14C10) antibody	Cell Signaling Technology, Danvers, MA, USA	Western blotting – 1:1000 dilution in 5% BSA in TBST overnight at 4°C
Trypan blue	Sigma-Aldrich, Gillingham, UK	Counting
Thiazolyl blue tetrazolium bromide solution (MTT)	Sigma-Aldrich, Gillingham, UK	MTT viability – 0.2% w/v in PBS
DMSO, 99.99%	Sigma-Aldrich, Gillingham, UK	
FITC Annexin V Apoptosis Detection Kit II	BD Biosciences, San Jose, CA, USA	Flow cytometry apoptosis analysis
Propidium iodide (PI), 95%	Acros Organics, Thermo Fisher Scientific, Geel, Belgium	Flow cytometry cell cycle analysis – 1mM in PBS; final concentration 60 µM/ml

1 mg/ml DNase and protease-free RNase A	Thermo Scientific, Loughborough, UK	Flow cytometry cell cycle analysis – final concentration 20 µg/ml

Supporting Methods: Section S1 - Transfection and growing stably transfected cell lines

A polymerase chain reaction product encoding amino acid residues 1–121 of PKC-δ, fused at the N-terminus with a myc epitope tag **(Figure S1)**, was subcloned into the NheI and NotI restriction sites of the mammalian expression vector pIRESneo2, generating the myc-δC2-pIRESneo2 plasmid. This vector has the myc-δC2 domain located upstream of the neomycin resistance marker on a single bicistronic mRNA driven by the CMV immediate early promoter.

EQKLISEEDLM^{1}APFLRIAFN^{10} SYELGSLQAE^{20} DEANQPFCAV^{30} KMKEALSTER^{40}
GKTLVQKKPT^{50}MYPEWKSTFD^{60}AHIYEGRVIQ^{70}IVLMRAAEEP^{80}VSEVTVGVSV^{90}
LAERCKKNNG^{100} KAEFWLDLQP^{110} QAKVLMSVQYF^{121}

Figure S1. Sequence of the PKC-δ C2 domain with a short myc tag (bold, red) attached to its N-terminus.

MDA-MB-468 and MCF-7 breast cancer cell lines were cultured and divided into two groups: one transfected with the empty pIRESneo2 vector, and the other with the myc-δC2-pIRESneo2 plasmid to overexpress the C2 domain of PKC-δ (myc-δC2). Both plasmids confer resistance to geneticin (G418), which was used to select transfected cells at 400 µM for MDA-MB-468 and 800 µM for MCF-7 cells.

For transfection, cells were seeded in 6-well plates at 5 x 10^5 cells/well 24 h before transfection. Media was changed to serum-free MEM 2 h before transfection.

Lipofectamine Transfection: 3 μg of DNA and 12.5 μL of Lipofectamine 2000 were added to 500 μL MEM, then incubated at RT for 20 min, and added to cells dropwise. Transfection media was replaced by selection media after 24 h.

Calcium phosphate transfection: 2.5 μg of DNA and 10 μL of 2.5 M CaCl2 were completed with water to 100 μL and incubated for 5 min at RT, then 100 μL 2x HBS pH 7.11 (280 mM NaCl, 1.5 mM Na2HPO4, 50 mM HEPES, 10 mM KCl, and 12 mM dextrose) were added, and the mixture was incubated for 30 min at 37 °C, then added to cells dropwise. Six h post-transfection, cells were subjected to a 1-min glycerol shock with 1 ml 15% glycerol in 1x HBS, then washed twice with PBS, before adding selection media.

After colony formation, cloning discs were used to pick and transfer the colonies into separate wells in a 24-well plate. Once confluent, cells were moved into T-75 flasks to establish stable cell lines, maintained in 300-350 μg/ml G418 for the course of their life.

Supporting Methods: Section S2 – Cell lysates and Western blotting

Cells were seeded at 5 x 10^5 cells/well in 6-well plates and were incubated until they reached confluency. Media, wash, and detached cells were harvested and centrifuged (4,000 rpm, 4 °C, 10 min). Pellets were dispersed with 100 μL of 1x RIPA (diluted in water) with protease and phosphatase inhibitors cocktail, then incubated on ice for 30 min. Attached cells were incubated with RIPA (300 μL, 30 min, on ice), then scraped and added to the pellets. Lysates were sonicated in water for 1 min, then back on ice for 1 min (repeated three times), then centrifuged (17,000 RCF, 4 °C, 20 min). Protein content in the supernatant was quantified using Pierce Bradford assay reagent following the manufacturer's instructions.

Protein expression was analyzed by Western blotting. Images acquired using the LI-COR imaging system (LI-COR Biosciences, Lincoln, Nebraska, USA) were processed in ImageJ v1.54. Band intensities were quantified as integrated density values. Myc-δC2 levels were normalized to GAPDH, which was used as a loading control.

Supporting Methods: Section S3 – Tetrazolium reduction viability assays (MTT assays)

Cells were seeded at 4 x 10^3 cells/well in 96-well plates and incubated for 24, 48, 72, and 144 h. To read, 50 μL of 0.2% (w/v) thiazolyl blue tetrazolium bromide solution dissolved in PBS was added to the wells. Plates were incubated for 3 h before aspirating the solution to add 50 μL of 99.99% DMSO. Plates were shaken in the dark for 1 min, and absorbance was measured at 570 nm. Readings were normalized to the viability measured at T_0 (3 h after seeding).

Supporting Methods: Section S4 – Apoptosis assays

FITC Annexin V Apoptosis Detection Kit II was used to assess cell apoptosis. Cells were seeded at a density of 1.1 x 10^5 cells/well in 24-well plates and incubated for 48 h. Media, cell wash, and harvested cells were all collected together and centrifuged (17,000 RCF, 4 °C, 10 min). Pellets were washed with 200 μL ice-cold PBS twice, then dried and resuspended in 100 μL of 1x annexin V binding buffer with 5 μL of FITC-annexin V and 5 μL of PI. After incubation in the dark for 15 min at RT, 400 μL of 1x binding buffer was added. Flow cytometry data were acquired using the Sony ID7000 Spectral Cell Analyzer and analyzed using Kaluza software. Gating was carried out as follows:

1. Debris exclusion by FSC-Area vs SSC-Area.
2. Singlet discrimination by SSC-Area vs SSC-Hight.

3. Apoptosis/necrosis analysis using PI-Area vs Annexin V-Area density plots. Quadrants were defined according to negative, Annexin V-only, and PI-only controls established during optimisation trials.

Supporting Methods: Section S5 – Cell cycle analysis

Cells were seeded and harvested as described for apoptosis. After incubation for 24, 48, and 72 h, cells were harvested and centrifuged. Pellets were washed twice with 200 µL PBS, air-dried, resuspended, and fixed with 100 µL of 70% ethanol on ice for 30 min, then centrifuged (17,000 RCF, 4 °C, 30 min). Resulting pellets were air-dried, washed twice with 200 µL PBS, then resuspended in 230 µL of PBS. Before reading, 15 µL of 1 mM 95% PI dissolved in PBS and 5 µL of 1 mg/ml DNase and protease-free RNase A were added at RT. Flow cytometry data were acquired using the Sony ID7000 Spectral Cell Analyzer. To analyze results using Kaluza, intact cells were first identified by gating on FSC-Area vs. SSC-Area to exclude debris. Single-cell events were subsequently isolated from doublets and aggregates by gating on the proportional relationship between PI signal area and height (PI-Area vs. PI-Height). The DNA content histogram (PI-Area) of the singlet population was then analyzed using the built-in Michael H. Fox cell cycle fitting model to quantify the percentage of cells in the G_0/G_1, S, and G_2/M phases of the cell cycle.

Supporting Methods: Section S6 – Cell imaging

Cells were seeded and harvested as described for apoptosis. Cells were incubated for 24, 48, and 72 h before harvesting. Following washing twice with 200 µL of cold PBS, pellets were resuspended in 50 µL ice-cold PBS for reading. Images of 10,000 cells/sample were taken at

three light intensities using the ImageStream Mk II. Images were analyzed with IDEAS 6.2 software using default gating to exclude debris and doublets, then generate plots of normalized cell count versus selected features (e.g., diameter, area, circularity). Statistical outputs included mean, median, mode, and event count.

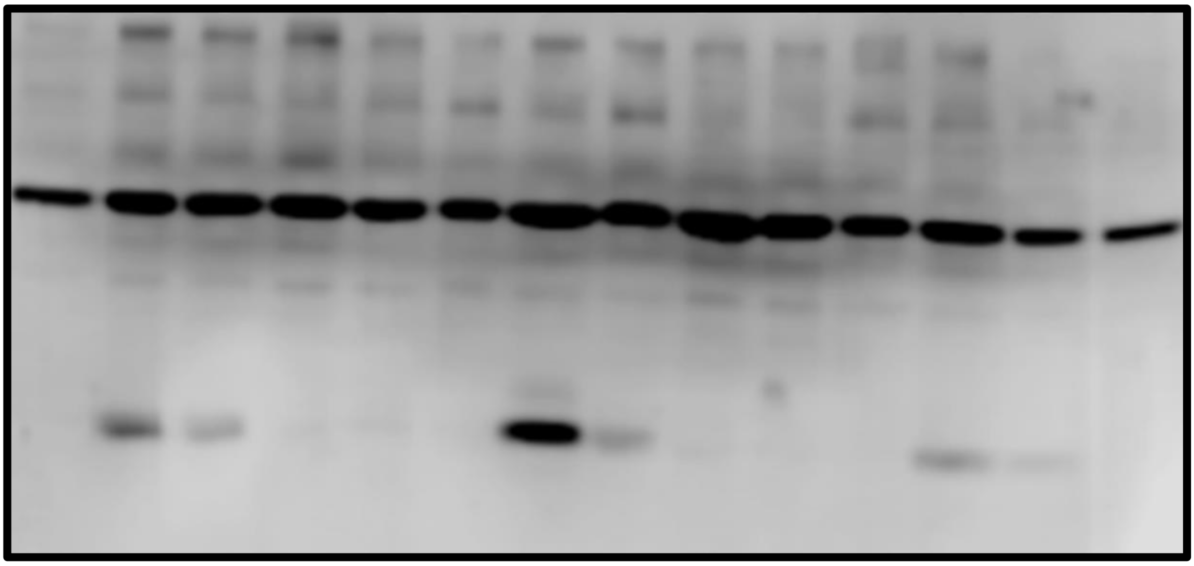

Figure S2. Uncropped Western blot corresponding to Figure 2 in the manuscript. Top bands represent GAPDH; bottom bands are myc-δC2. Lanes: 2-MDA-MB-468/δC2.1, 3-MDA-MB-468/δC2.2, 4-MDA-MB-468/Vector.1, 5-MDA-MB-468/Vector.2, 7-MCF-7/δC2.1, 8-MCF-7/δC2.2, 9-MCF-7/Vector.1, 10-MCF-7/Vector.2.

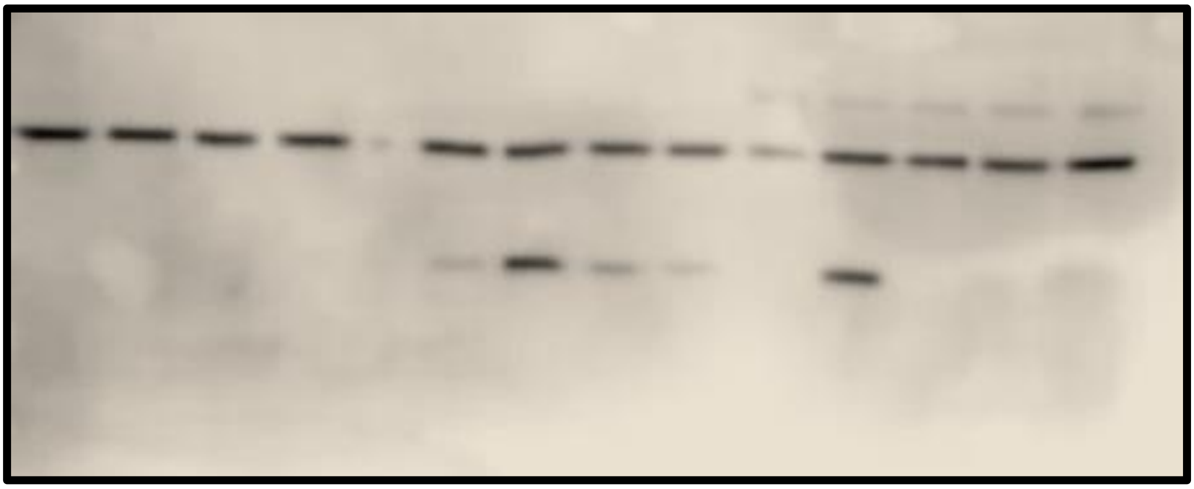

Figure S3. Uncropped Western blot corresponding to Figure 4 in the manuscript. Top bands represent GAPDH; bottom bands are myc-δC2. Lanes: 1-MDA-MB-468/δC2.1, 2-MDA-MB-468/δC2.2, 3-MDA-MB-468/Vector.1, 4-MDA-MB-468/Vector.2, 11-MCF-7/δC2.1, 12-MCF-7/δC2.2, 13-MCF-7/Vector.1, 14-MCF-7/Vector.2.

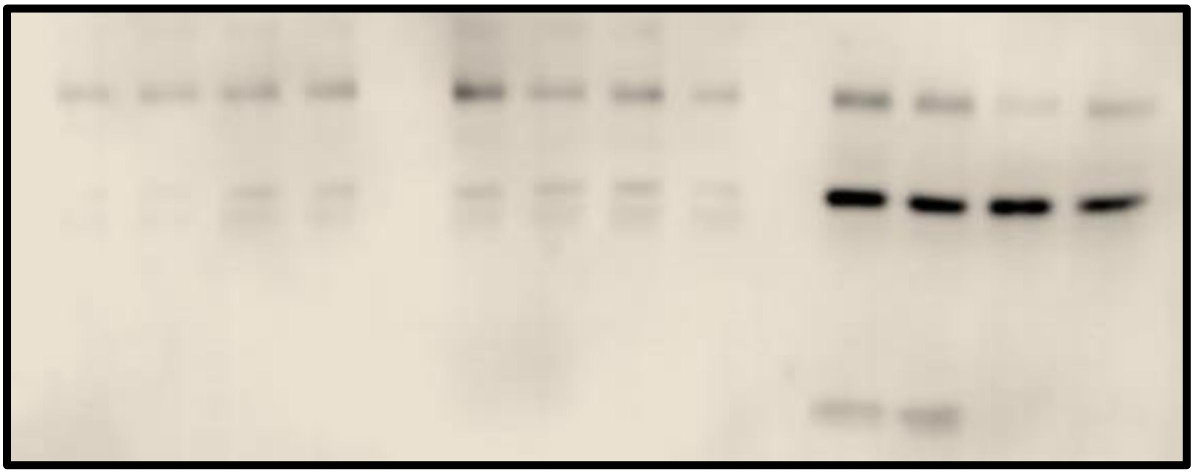

Figure S4. Uncropped Western blot corresponding to Figure 5 (MDA-MB-468) in the manuscript. Middle bands represent GAPDH; bottom bands are myc-δC2. Lanes: 9-MDA-MB-468/δC2.1, 10-MDA-MB-468/δC2.2, 11-MDA-MB-468/Vector.1, 12-MDA-MB-468/Vector.2.

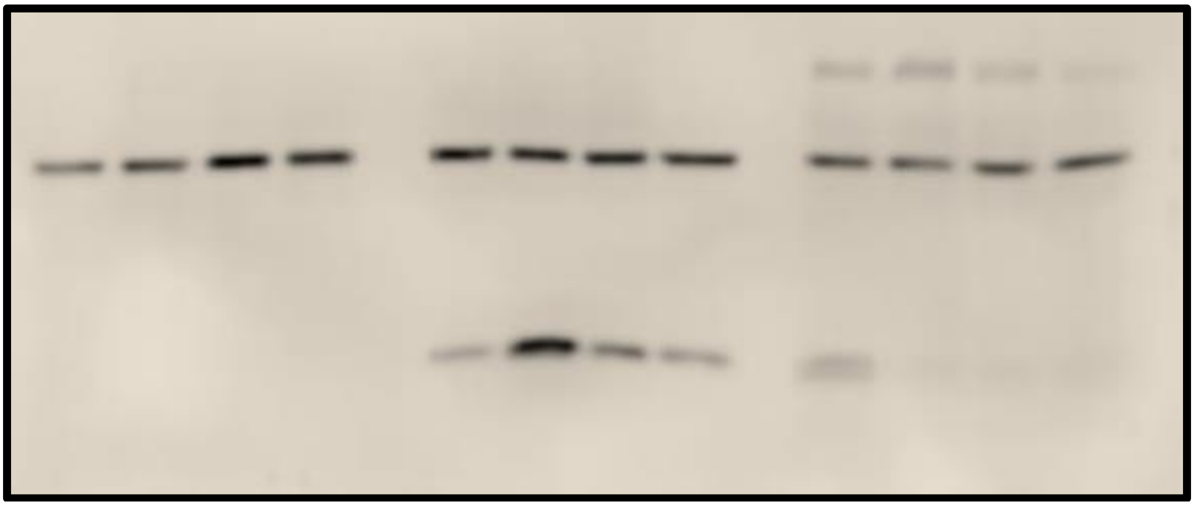

Figure S5. Uncropped Western blot corresponding to Figure 5 (MCF-7) in the manuscript. Middle bands represent GAPDH; bottom bands are myc-δC2. Lanes: 9-MCF-7/δC2.1, 10-MCF-7/δC2.2, 11-MCF-7/Vector.1, 12-MCF-7/Vector.2.

Present Address

† Department of Clinical Pharmacy, Faculty of Pharmacy, Jordan University of Science and Technology, P.O. Box 3030, Irbid 22110, Jordan.

References

1. Scott, H. E. Pkc-Δ, Its C2 Domain and Breast Cancer Cell Lines. PhD Thesis, University of Nottingham, 2012.
